# Therapeutic and Diagnostic Landscape of Diabetic Neuropathy: A Systematic Review of Clinical Studies

**DOI:** 10.7759/cureus.102825

**Published:** 2026-02-02

**Authors:** Jay Prakash S Rajput, Rahul Deb, Sajidali S Saiyad, Zayan Jamal, Santosh Kumar Sah, Alkeshkumar R Vara, Nadia Sandhu, Dimpal Rochlani, Tanzila A Saiyed, Arin A Pathan, Ashok Sagar, Tejal Virola

**Affiliations:** 1 Physiology, Shri Gorakshnath Medical College Hospital and Research Centre, Gorakhpur, IND; 2 Pharmacology, Maharishi Markandeshwar (MM) Institute of Medical Sciences and Research, Ambala, IND; 3 Physiology, Pacific Medical College and Hospital, Pacific Medical University, Udaipur, IND; 4 Internal Medicine, Amrita School of Medicine, Amrita Vishwa Vidyapeetham, Kochi, IND; 5 Physiology, Krishna Mohan Medical College and Hospital, Mathura, IND; 6 Physiology, Nootan Medical College and Research Centre, Visnagar, IND; 7 Clinical Sciences, Washington University of Health and Science, San Pedro, BLZ; 8 Biochemistry, GS Medical College and Hospital, Pilkhuwa, IND; 9 Pediatrics, Samarkand State Medical University, Samarkand, UZB; 10 Family Medicine, Samarkand State Medical University, Samarkand, UZB; 11 Forensic Medicine, Santosh Medical College and Hospital, Ghaziabad, IND; 12 Mental Health Nursing, Institute of Nursing Sciences, Shri GH Patel School of Nursing, Bhaikaka University, Karamsad, IND

**Keywords:** antioxidant therapy, biomarkers, diabetic autonomic neuropathy (dan), diabetic lumbosacral radiculoplexus neuropathy, diabetic neuropathy, diabetic sensorimotor polyneuropathy, disease-modifying therapy, multimodal therapy, refractory neuropathic pain, regenerative medicine

## Abstract

Diabetic neuropathy (DN) is one of the most prevalent complications of diabetes mellitus, affecting up to half of patients and contributing to disability, poor quality of life, and the risk of foot ulceration. Despite extensive research, its heterogeneous manifestations and complex pathophysiology continue to challenge timely diagnosis and effective treatment. This systematic review aimed to synthesize recent clinical evidence on diagnostic and therapeutic strategies for DN.

A comprehensive search of PubMed, EMBASE, CENTRAL, and Web of Science identified 76 eligible clinical studies published between 2020 and 2025, including randomized controlled trials, observational studies, and case reports. Data extraction and risk-of-bias assessment were performed according to PRISMA guidelines.

Pharmacological agents, particularly pregabalin, duloxetine, and α-lipoic acid, demonstrated the most consistent efficacy, with significant pain reduction and improvements in nerve conduction velocity. Neuromodulation with high-frequency spinal cord stimulation provided sustained pain relief in refractory cases, while structured, exercise-based rehabilitation improved gait velocity and balance. Advanced wound care strategies, such as platelet-rich plasma dressings and bioengineered skin substitutes, accelerated ulcer healing. Complementary therapies (e.g., acupuncture and balneotherapy) and emerging biologics (e.g., gene- and cell-based interventions) showed preliminary promise but require further validation.

Nerve conduction studies and validated scoring instruments remain the most reliable diagnostic tools, with biomarker- and microvascular-based measures emerging as valuable adjuncts. Current evidence underscores the value of integrating pharmacological, device-based, and rehabilitative strategies, while highlighting critical gaps in small-fiber and autonomic neuropathies. Robust, multicenter trials are needed to establish disease-modifying therapies and optimize comprehensive care pathways for DN.

## Introduction and background

Diabetes mellitus is a metabolic disorder that frequently affects the peripheral nervous system [1]. Diabetic neuropathy (DN) is one of the most common chronic complications of diabetes mellitus, affecting approximately 30%-50% of individuals with long-standing disease; indeed, it has been reported that at least half of all people with diabetes develop neuropathy over time, contributing substantially to morbidity, disability, and the global healthcare burden [2]. While diabetes is a major cause, neuropathy may also result from other factors, such as excessive alcohol consumption, vitamin deficiencies, injuries, infections, and inflammatory conditions [3-5]. In patients with poorly controlled glycemic levels, secondary complications can arise due to the accumulation of metabolites from the polyol pathway. These metabolites increase oxidative stress and reduce nerve glutathione content, thereby contributing to nerve damage [6].

DN represents a heterogeneous spectrum of neuropathic disorders, each requiring a personalized approach to management. This spectrum includes neuropathy associated with impaired glucose tolerance and hyperglycemia [7]; generalized symmetrical neuropathies - most commonly distal symmetric sensorimotor polyneuropathy [8,9] - acute painful neuropathy [10], small fiber neuropathy, autonomic neuropathy [11], acute motor axonal neuropathy [12], focal and multifocal neuropathies, including cranial and thoracolumbar involvement [13-15], diabetic lumbosacral radiculoplexus neuropathy (also known as Bruns-Garland syndrome or diabetic amyotrophy) [16], superimposed chronic inflammatory demyelinating polyneuropathy [17], and hypoglycemic neuropathy [18]. Among these, distal symmetric sensorimotor polyneuropathy is the most common manifestation, accounting for the majority of cases of DN [8].

Diagnosis and grading of DN are based on structured clinical assessment, validated clinical scoring systems, and electrophysiological evaluation, in line with widely accepted clinical practice and consensus-based recommendations. Diagnostic modalities include nerve conduction studies and electromyography, along with adjunctive investigations such as cerebrospinal fluid analysis [19], nerve biopsy, and neuroimaging techniques, including computed tomography, magnetic resonance imaging, and positron emission tomography; additional assessments may involve electroencephalography and evoked potentials when clinically indicated [20]. Both acute and chronic diabetes predispose individuals to peripheral arterial disease and sensory neuropathy, particularly involving the feet. Peripheral nerve damage leads to autonomic dysfunction, with reduced sweating, skin dryness, and diminished pain perception, allowing minor injuries to go unnoticed and significantly increasing the risk of diabetic foot ulceration [21].

Multiple therapeutic approaches are available for the management of DN, encompassing pharmacological treatment, lifestyle modification, physical rehabilitation, and wound care. First-line pharmacological agents for painful DN include pregabalin (150-600 mg/day), duloxetine (60-120 mg/day), and amitriptyline (25-75 mg/day), selected according to efficacy, comorbidities, and tolerability. Adjunctive therapies, such as α-lipoic acid (600 mg/day), may provide additional neuroprotective benefit, while combination pharmacotherapy or neuromodulation strategies - including high-frequency spinal cord stimulation (SCS) - may be considered in refractory cases. Non-pharmacological measures - including regular physical activity, nutritional interventions, physical therapy, and supportive devices such as braces, orthotics, and walking aids - play a crucial role in improving function and quality of life. Management of diabetic foot complications involves multidisciplinary care, with wound debridement, advanced dressings, negative pressure wound therapy, bioengineered skin substitutes, and selected adjuvant therapies, such as platelet-rich plasma or granulocyte colony-stimulating factor, when indicated [22].

Given the high incidence of secondary complications and the progressive nature of DN, there is an urgent need for timely diagnosis and effective interventions to prevent progression and disability. The primary objective of this systematic review is to synthesize contemporary clinical evidence published within the last five years on diagnostic and therapeutic strategies for DN, while referencing key seminal studies published earlier to provide foundational context and support interpretation of recent advances.

## Review

Methods

Study Design

This systematic review was conducted in accordance with the PRISMA 2020 guidelines. The review protocol was not prospectively registered in PROSPERO or any other database, which is a limitation. The absence of registration was due to the retrospective nature of this review and time constraints during study initiation. Future systematic reviews will aim for prospective registration to enhance methodological transparency and reduce bias.

Eligibility Criteria

Eligibility criteria were defined according to the PICO framework: (I) Population: patients with DN, including all subtypes (sensorimotor, autonomic, focal/multifocal, and small fiber). (II) Intervention: any therapeutic (pharmacological, surgical, lifestyle, or alternative) or diagnostic strategy for DN. (III) Comparison: placebo, standard care, or other active interventions. (IV) Outcomes: the primary outcome was pain reduction, measured by validated scales (Visual Analog Scale (VAS) or Numerical Rating Scale (NRS)) at 12-16 weeks. Secondary outcomes included functional outcomes (e.g., mobility and balance), neurophysiological parameters (nerve conduction velocity), and complication-related outcomes (ulcer healing and amputation rates). Outcomes were harmonized to common timepoints, where feasible. Study eligibility and outcomes were defined using the PICO framework (Table 1).

**Table 1:** PICO framework of the study PICO: Population, Intervention, Comparison, Outcomes; MeSH: Medical Subject Headings; CENTRAL: Cochrane Central Register of Controlled Trials

Component	Details
Databases searched	PubMed, EMBASE, Cochrane Central Register of Controlled Trials (CENTRAL), Web of Science
Time frame	January 2000 - March 2025 (final inclusion focused on Jan 2020 - Mar 2025)
Study types included	Randomized controlled trials, prospective and retrospective cohort studies, clinical studies
Study types excluded	Animal studies, narrative/systematic reviews, conference abstracts, preprints, letters, non-clinical studies
Key concepts	Diabetic neuropathy, diagnosis, therapy, management
Main search terms	“diabetic neuropathy”, “diabetic peripheral neuropathy”, “painful diabetic neuropathy”, “diabetic autonomic neuropathy”, “diabetic polyneuropathy”
Intervention/diagnosis terms	“therapy”, “treatment”, “intervention”, “management”, “rehabilitation”, “diagnosis”, “diagnostic”, “biomarker”, “screening”
Boolean structure	(Neuropathy terms) AND (therapy/diagnosis terms)
Example PubMed strategy	(“Diabetic Neuropathies”[MeSH] OR diabetic neuropathy OR painful diabetic neuropathy OR diabetic autonomic neuropathy) AND (therapy OR treatment OR intervention OR diagnostic OR biomarker OR screening)
Records identified	8,315
After deduplication & date filter	1,478
Final studies included	76

We included randomized controlled trials (RCTs), prospective cohort studies, and retrospective cohort studies that reported clinically relevant outcomes in patients with DN. The following were excluded from the primary analysis: non-clinical studies, animal or veterinary research, narrative reviews, systematic reviews, conference abstracts, preprints, letters, adaptive clinical trials, and any studies lacking clinical outcome data. Only articles published within the last five years were eligible for detailed evaluation. Case reports, case series, and pilot non-comparative studies were not included in the quantitative synthesis.

Information Sources and Search Strategy

A comprehensive search was conducted in PubMed, EMBASE, Cochrane Central Register of Controlled Trials (CENTRAL), and Web of Science. The search covered studies published between January 2000 and March 2025. The following keywords and Boolean operators were used: “diabetic neuropathy” OR “diabetic peripheral neuropathy” OR “diabetic autonomic neuropathy” OR “painful diabetic neuropathy” OR “diabetic polyneuropathy” AND “therapy” OR “treatment” OR “intervention” OR “management” OR “diagnosis” OR “diagnostic”.

The full PubMed search syntax is provided as follows: ("Diabetic Neuropathies"[MeSH] OR "diabetic neuropathy" OR "diabetic peripheral neuropathy" OR "painful diabetic neuropathy" OR "diabetic autonomic neuropathy" OR "diabetic polyneuropathy" OR "neuropathic pain in diabetes") AND ("therapy"[MeSH] OR therap* OR treatment* OR management OR intervention* OR rehabilitation OR pharmacological OR nonpharmacological OR diagnostic OR diagnosis OR biomarker* OR screening) AND ("2000/01/01"[Date - Publication] : "2025/03/31"[Date - Publication]).

**Figure 1:**
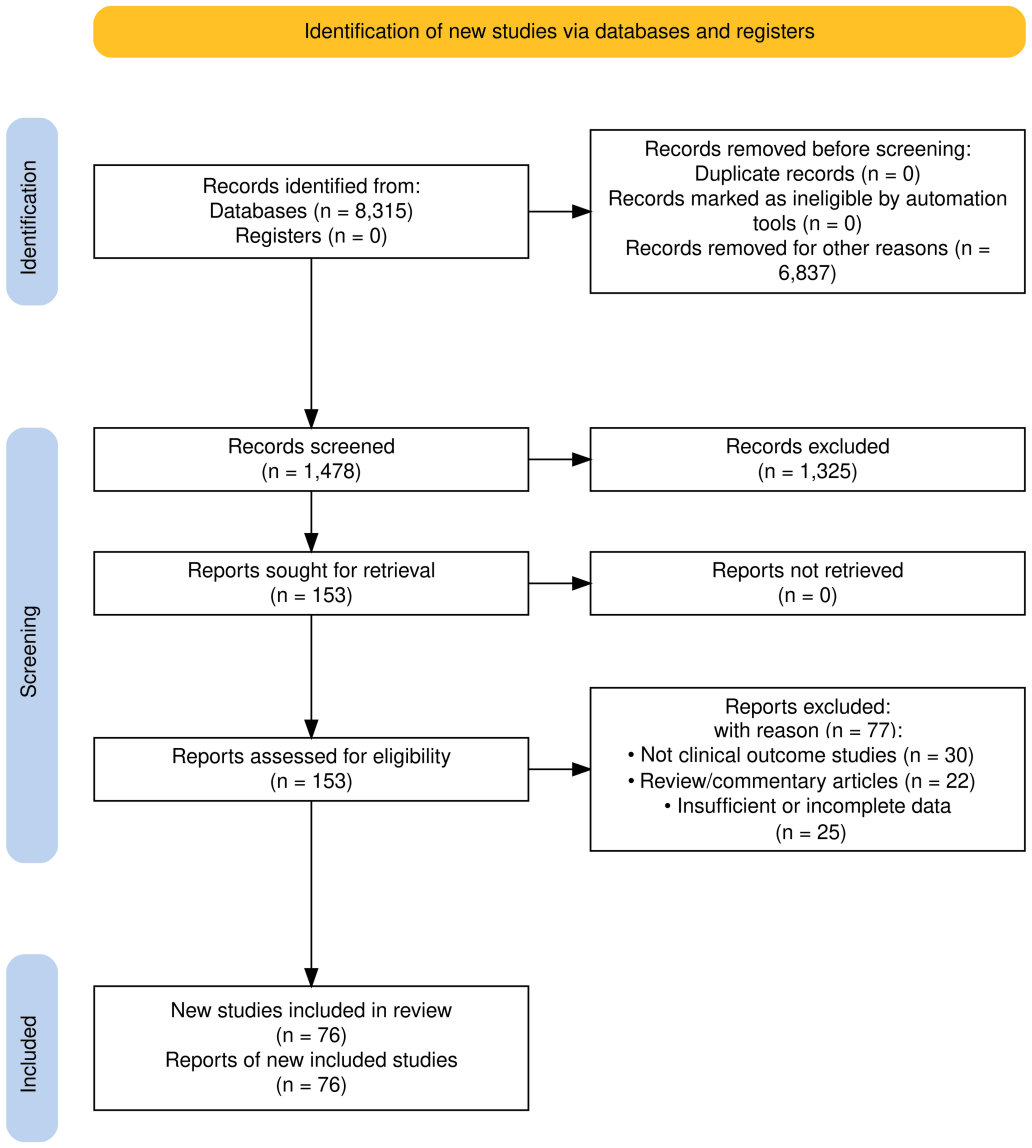
PRISMA flow diagram of the study selection process PRISMA: Preferred Reporting Items for Systematic Reviews and Meta-Analyses

The search retrieved 8,315 records. After deduplication and restricting to January 2020-March 2025, 1,478 records remained. Of these, 1,325 were excluded at the title/abstract stage, and 74 at the full-text stage, leaving 76 included studies. A detailed PRISMA 2020 flow diagram, with reasons for exclusion, is shown in Figure 1. The detailed search strategy and information sources are summarized in Table 2.

**Table 2:** Search strategy and information sources PICO: Population, Intervention, Comparison, Outcomes; VAS: Visual Analog Scale; NRS: Numerical Rating Scale; NCV: Nerve Conduction Velocity

Element	Description
Population (P)	Patients with diabetic neuropathy
Intervention (I)	Any therapeutic (pharmacological, neuromodulation, rehabilitation, lifestyle, complementary) or diagnostic strategy for diabetic neuropathy
Comparison (C)	Placebo, standard care, sham intervention, or alternative active interventions
Outcomes (O)	Primary: Pain reduction measured by validated scales (VAS/NRS) at 12-16 weeks; Secondary: Functional outcomes (mobility, balance), neurophysiological measures (NCV), and complication-related outcomes (ulcer healing, amputation rates)

Study Selection

Two independent reviewers screened titles and abstracts for relevance. Full-text articles were then assessed against eligibility criteria. Disagreements were resolved by consensus or consultation with a third reviewer.

Data Extraction

Data were extracted using a standardized data extraction form. The following variables were collected: author, year of publication, country, study design, sample size, neuropathy subtype, intervention details, comparator, outcomes measured, follow-up duration, and main findings. Articles were categorized into therapeutic studies, diagnostic studies, and case reports, with further stratification based on intervention type and neuropathy subtype. Sample sizes and quantitative outcome measures were extracted directly from original publications; where such data were not explicitly reported, this was clearly indicated to maintain transparency.

Risk of Bias Assessment

Risk of bias was independently assessed by two reviewers. RCTs were evaluated using the Cochrane RoB 2 tool, while non-randomized studies were assessed with the ROBINS-I tool. Discrepancies were resolved by consensus. Results of the risk of bias assessment are summarized in the Results section and discussed in detail.

Data Synthesis

Owing to substantial clinical and methodological heterogeneity across trials (differences in populations, outcome scales, timepoints, and intervention protocols), we prespecified a narrative synthesis approach in line with PRISMA 2020. We summarized the direction and consistency of effects, study quality, and clinical relevance without quantitative pooling. When comparable, we report the range of effects and note the number of studies favoring the intervention versus the comparator. Sensitivity judgments considered the risk of bias, and outcome measurement differences.

Results

Search Results and Study Characteristics

The initial search identified 8,315 articles published between 2000 and 2025. After applying eligibility criteria and removing duplicates, 1,478 studies remained for title and abstract screening. Following full-text review, 76 studies met the inclusion criteria (Figure 1). The included studies consisted of RCTs, observational studies, and case reports. The majority investigated therapeutic interventions (76%), whereas 16% evaluated diagnostic strategies, and 8% were case-based clinical reports.

**Figure 2:**
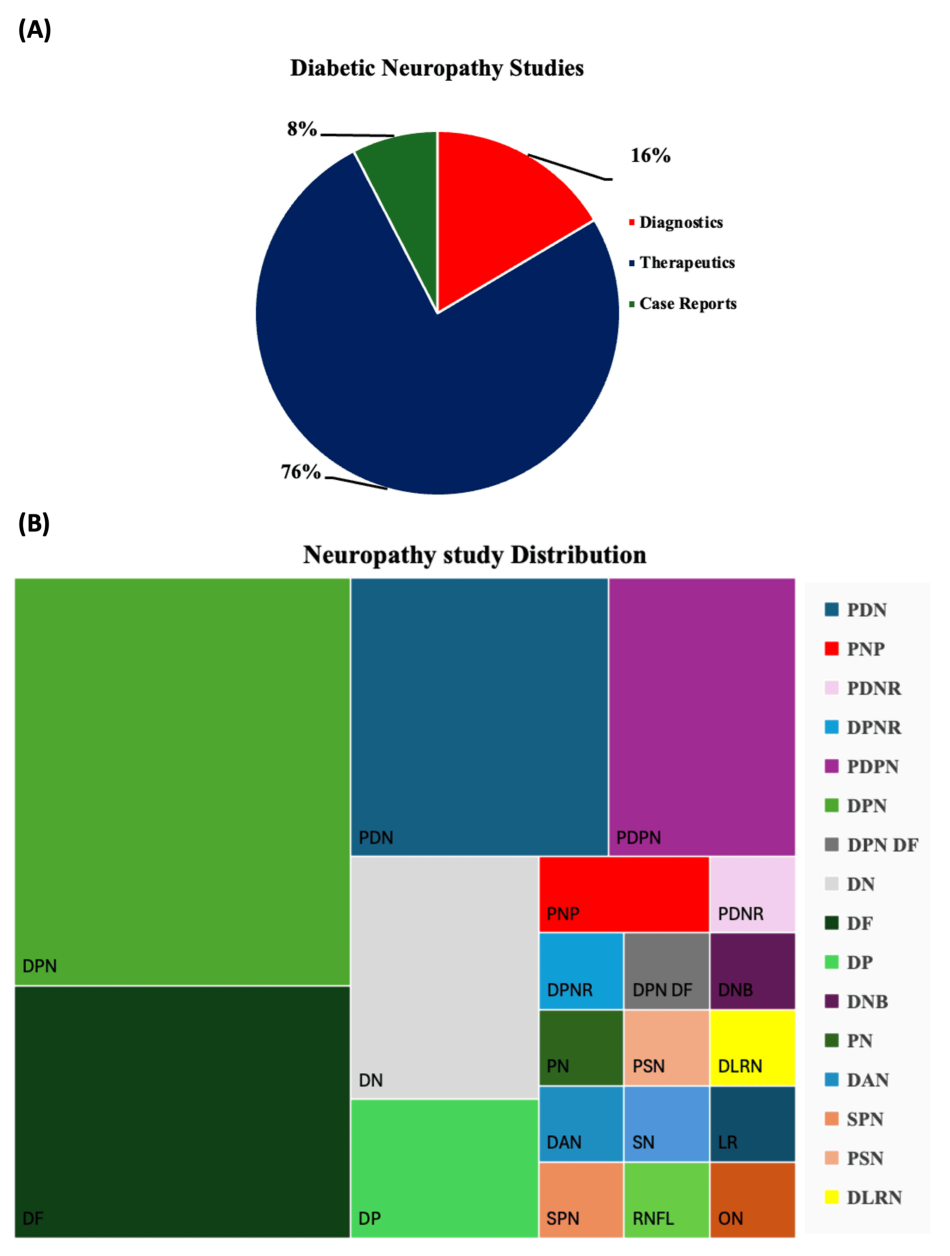
Systematic analysis of diabetic neuropathy studies (2020-2025) (A) Distribution of diabetic neuropathy studies by thematic focus. (B) Distribution of Neuropathy subtypes. Data represent the number of included studies (documents), not patient counts or pooled prevalence. Each study was classified once according to its primary thematic focus or predominant neuropathy subtype. DPN: Diabetic Peripheral Neuropathy; DF: Diabetic Foot; DN: Diabetic Neuropathy; PDN: Painful Diabetic Neuropathy; DP: Diabetic Polyneuropathy; PNP: Peripheral Neuropathic Pain; PDNR: Painful Diabetic Peripheral Neuropathy Refractory; DPNR: Refractory Diabetic Peripheral Neuropathy; PDPN: Diabetes-Related Peripheral Neuropathy; DPN DF: Diabetic Foot; DNB: Diabetic Neurogenic Bladder; PN: Peripheral Neuropathy; DAN: Diabetic Autonomic Neuropathy; SPN: Sensory Polyneuropathy; PSN: Peripheral Sensorimotor Neuropathy; DLRN: Diabetic Lumbosacral Radiculoplexus Neuropathy; SN: Sensory Neuropathy; RNFL: Peripapillary Retinal Nerve Fiber Layer; LR: Painless Nondiabetic Lumbosacral Radiculoplexus; ON: Optic Neuropathy

Table 3 summarizes the characteristics of the included studies, detailing author, year, study design, sample size, neuropathy subtype, intervention, comparator, and key outcomes. The distribution of the 76 included studies, by their primary thematic focus and the specific neuropathy subtypes they investigated, is presented in Figure 2. Pharmacological interventions constituted the largest thematic category, while studies on DN were the most prevalent.

**Table 3:** Summary of clinical evidence on interventions, diagnostics, and rehabilitation strategies in diabetic neuropathy Data are expressed as n, %, or mean ± SD, unless otherwise specified. Statistical significance was defined as p < 0.05 (highly significant at p < 0.001). Where reported in the original studies, the test statistic is indicated (t value for t-tests, χ² for chi-square tests, and F value for ANOVA); when unavailable, this is denoted as “Not reported.” Evidence type reflects the primary therapeutic or methodological category of each study. * Sample size retrieved from published abstract or article; NR: sample size not reported in the accessible abstract/summary; NA: not applicable (e.g., study protocol, case report, review). Protocol studies: Trial protocols or planned studies for which participant enrollment was ongoing or not yet completed; reported sample sizes represent planned enrollment, where stated. DPN: Diabetic Peripheral Neuropathy; PDPN: Painful Diabetic Peripheral Neuropathy; DN: Diabetic Neuropathy; DF: Diabetic Foot; QoL: Quality of Life; VAS: Visual Analog Scale; NCV: Nerve Conduction Velocity; HRV: Heart Rate Variability; COP: Center of Pressure; NCS: Nerve Conduction Study; TENS: Transcutaneous Electrical Nerve Stimulation; NMES: Neuromuscular Electrical Stimulation; rTMS: Repetitive Transcranial Magnetic Stimulation; NPWT: Negative Pressure Wound Therapy; PRP: Platelet-Rich Plasma; HGF: Hepatocyte Growth Factor; VEGFA: Vascular Endothelial Growth Factor A; TGF-β: Transforming Growth Factor-β

Sr. No.	Author(s), Year	Study Design	Reported Sample Size	Neuropathy Subtype	Intervention	Comparator	Key Outcomes
1	Kamiya et al. (2020) [23]	RCT	82 [*]	General DN	Point-of-Care NCS Device	Standard NCS	Predicts polyneuropathy severity
2	Sun et al. (2020) [24]	RCT	120	Painful DPN	Pregabalin	Placebo	VAS pain ↓ 2.1 points; improved sleep
3	Barzilay et al. (2021) [25]	Cross-sectional	865 [*]	DPN with autonomic involvement	Biomarker assessment (HGF, VEGFA, TGF-β)	Controls	Cognition associated with neuropathy severity
4	Ng et al. (2020) [26]	RCT	80 [*]	Diabetic neuropathy	Tocotrienol-rich Vitamin E (Tocovid)	Placebo	Improved neuropathic pain outcomes
5	Ng et al. (2022) [27]	Cross-sectional	92 [*]	DPN with PAD risk	Toe-brachial index; ASTP assessment	Reference values	Diagnostic thresholds established
6	Elghazaly et al. (2023) [28]	Diagnostic study	NR	DPN with ulcer risk	Skin perfusion pressure; transcutaneous O₂ pressure	Ulcer healing outcomes	Prognostic value for ulcer healing
7	Guo L et al. (2024) [29]	RCT Phase 3	1,657 [*]	DPN	Acetyl-L-carnitine HCl	Control	Reduced pain; improved NCV
8	Didangelos et al. (2024) [30]	RCT	139 [*]	DPN	PEA + SOD + α-lipoic acid + Vitamins B12, B1	Placebo	Pain reduction; improved functional outcomes
9	Lin L et al. (2022) [31]	RCT	114 [*]	DPN	1064-nm Infrared Laser (adjuvant)	Standard care	Adjuvant therapy for DPN pain
10	Kostopoulos et al. (2025) [32]	Comparative	NR	DPN	NMES vs TENS	Comparative analysis	Physical therapy adjunct; mixed results
11	Deng et al. (2020) [33]	RCT	65	Autonomic DN	Acupuncture	Sham acupuncture	Pain scores ↓ 1.4 VAS; improved HRV
12	Dietzel et al. (2021) [34]	RCT Protocol	NA (Protocol)	DPN	Acupuncture (ACUDPN trial)	Placebo acupuncture	Multicenter protocol for DPN acupuncture
13	Orlando et al. (2024) [35]	RCT (Crossover)	28 [*]	DPN	Vibrating insoles	Standard insoles/control	Improvements in dynamic balance; gait quality ↑
14	Han et al. (2020) [36]	RCT Protocol	NA (Protocol)	Diabetic neurogenic bladder	Electroacupuncture	Sham electroacupuncture	Protocol for autonomic neuropathy
15	Pérez Hernández et al. (2024) [37]	RCT Protocol	NA (Protocol)	Diabetic polyneuropathy	Electroacupuncture	Sham electroacupuncture	Multicenter RCT protocol
16	Dogaru et al. (2025) [38]	Prospective observational	45 [*]	DPN	Balneotherapy (spa therapy)	No intervention	Pain ↓; walking ↑; function ↑
17	Tesfaye et al. (2022) [39]	RCT (Crossover)	140 [*]	PDPN	Amitriptyline + Pregabalin vs alternatives	Sequential pharmacological	OPTION-DM trial; multiple agents effective
18	Wallace et al. (2020) [40]	RCT secondary analysis	28 (Sub-analysis)	PDPN	THC (plasma level analysis)	Placebo	THC plasma levels associated with pain reduction
19	Maksymowicz et al. (2024) [41]	Case report	NA (Case Report)	PDPN with opioid hyperalgesia	Multimodal management	Historical comparison	Comprehensive pain management
20	Okdahl et al. (2021) [42]	RCT Protocol	NA (Protocol)	Diabetic autonomic neuropathy	Transcutaneous vagal nerve stimulation (DAN-VNS)	Sham stimulation	Autonomic neuropathy protocol; GI focus
21	Zhai et al. (2022) [43]	RCT	128 [*]	Type 2 DPN	Acupoint injection (Zusanli ST36)	Control	DTI imaging shows improvement markers
22	Dhanapalaratnam et al. (2024) [44]	Cohort	90	Sensorimotor DN	Structured exercise program	No intervention	Gait speed ↑; NCV ↑; reduced falls
23	Tay et al. (2021) [45]	RCT	80 [*]	DPN	Moxibustion	Control/standard	Pain relief efficacy in diabetic neuropathy
24	Jafarzadeh et al. (2023) [46]	RCT	60 [*]	PDPN	Memantine	Placebo	Neuropathic pain reduction
25	Tiecke et al. (2022) [47]	RCT proof of concept	67 [*]	PDPN	NRD.E1 (non-opioid agent)	Placebo	Novel analgesic agent; pain reduction
26	Lu et al. (2022) [48]	RCT	120 [*]	PDPN	Xiaoketongbi formula	Pregabalin	Traditional Chinese medicine vs standard
27	Pickering et al. (2022) [49]	RCT	67 [*]	PDPN	Palmitoylethanolamide (PEA)	Placebo	PDPN pain reduction; safety profile
28	Gálvez et al. (2025) [50]	RCT (phase 2)	209 [*]	PDPN + chronic postsurgical pain	E-52862 (sigma-1 antagonist)	Placebo	Novel mechanism pain agent
29	Jatuten et al. (2023) [51]	RCT double-blind	88 [*]	PDPN	Topical Zingiber cassumunar	Placebo	Herbal topical therapy
30	Lipone et al. (2020) [52]	RCT pilot	40 [*]	PDPN (on gabapentin)	Low-dose trazodone (adjunctive)	Placebo	Adjunctive therapy; CNS effects
31	Pop-Busui et al. (2024) [53]	RCT proof of concept	319 [*]	PDPN (refractory)	LX9211 (sodium channel blocker)	Placebo	RELIEF-DPN 1 trial; novel non-opioid
32	Zhang et al. (2022) [54]	RCT protocol	210 (Planned) [*]	PDPN	Mudan Granules (traditional)	Placebo	Traditional medicine protocol
33	Petersen et al. (2022) [55]	RCT	450	Refractory PDPN	High-frequency (10-kHz) SCS	Medical management	Durability 12 months; sustained relief
34	Petersen et al. (2021) [56]	RCT	216 [*]	PDPN (refractory)	10-kHz spinal cord stimulation	Standard SCS/medical	Superior pain relief vs conventional
35	Gonçalves et al. (2021) [57]	RCT	18 [*]	DPN (ischemic rest pain)	TENS	Sham TENS	Peripheral vascular disease pain management
36	Zhuang et al. (2024) [58]	RCT protocol (three-armed)	198 (Planned) [*]	DPN	Electro-acupuncture vs sham acupuncture	Sham + control	Three-arm design; rigorous protocol
37	Rao et al. (2023) [59]	RCT multicenter	44 [*]	PDPN	Repetitive transcranial magnetic stimulation (rTMS)	Sham rTMS	Pain relief; brain stimulation
38	Yang et al. (2022) [60]	Study	50 [*]	DPN (acute response)	Short-term repetitive TMS	Control stimulation	Acute neuropathic pain response
39	Kessler et al. (2021) [61]	RCT	150	General DN	α-lipoic acid	Placebo	Improved NCV (+3.8 ms); ↓ oxidative stress
40	Chuar et al. (2021) [62]	RCT double-blind	80 [*]	Type 2 DM with neuropathy	Tocotrienol-rich vitamin E (Tocovid)	Placebo	Improved NCV; Phase II trial
41	Won et al. (2020) [63]	RCT noninferiority	114 [*]	PDPN	α-linolenic acid vs α-lipoic acid	Comparator active therapy	12-week double-placebo assessment
42	Esposito et al. (2021) [64]	RCT double-blind	126 (diabetic subset) [*]	Neuropathic pain (diabetes subset)	α-lipoic acid (oral)	Placebo	Pain reduction subgroup analysis
43	Seidel et al. (2020) [65]	RCT	70	DF ulcers	PRP dressings	Standard dressings	57% complete ulcer closure at 12 weeks
44	Simman et al. (2024) [65]	RCT clinical study	40 [*]	Diabetic foot ulcers	Multimodal wound matrix (novel product)	Standard dressings	Accelerated healing; ulcer closure
45	Slivnik et al. (2024) [66]	RCT placebo-controlled	40 [*]	Chronic diabetic foot ulcers	Chitosan gel (CHITOWOUND)	Placebo	Novel biomaterial; wound efficacy
46	Zhong et al. (2024) [67]	RCT	46 [*]	Diabetic foot ulcers	NPWT + antibiotic-loaded bone cement	Standard NPWT	Enhanced healing with combination
47	Huang et al. (2021) [68]	RCT	68 [*]	Diabetic foot ulcers	Macrophage-regulating drug (novel)	Placebo	Immune modulation; wound healing
48	Lipsky et al. (2024) [69]	RCT phase 1b	42 [*]	Diabetic foot infections	Topical pravibismane (adjunctive)	Placebo	Phase 1b safety/efficacy
49	Wang et al. (2024) [70]	Study	NR	Diabetic foot ulcers	Electrospun PLCL + porcine fibrinogen scaffold	Standard dressings	Bioengineered scaffold; regeneration
50	Basiri et al. (2022) [71]	Intervention study	60 [*]	Diabetic foot ulcers (obese/overweight)	Nutrition intervention	Standard care	HbA1c ↓; body composition improvements
51	Znica-García et al. (2024) [72]	Observational	106 [*]	Type 2 DPN	Dietary habit assessment	Reference group	Dietary influence on foot risk
52	Cruvinel-Júnior et al. (2024) [73]	RCT	224 [*]	DPN	Web-based foot-ankle exercise (affordable)	Usual care	Effective digital rehabilitation
53	Cruvinel-Júnior et al. (2022) [74]	Proof-of-concept	36 [*]	DPN	Internet-based foot-ankle therapeutic exercise	No intervention	Gait biomechanics; clinical outcomes
54	Ferreira et al. (2024) [75]	RCT	80 [*]	DPN (ulcer risk)	Web-based foot-ankle exercise program	Usual care	Ulcer risk reduction; efficacy
55	Ferreira et al. (2024) [75]	RCT	76 [*]	DPN with gait impairment	Structured exercise + rehabilitation	Usual care	Functional mobility; ulcer risk ↓
56	Monteiro et al. (2023) [76]	RCT (secondary outcomes)	80 [*]	DPN	Foot-ankle exercise program	Control group	Kinematics ↑; plantar pressure ↓
57	Monteiro et al. (2022) [77]	RCT	80 [*]	DPN	Foot-ankle therapeutic exercise	No intervention	Gait speed ↑ (primary outcome)
58	Brown et al. (2021) [78]	Observational/Imaging	19 [*]	DPN	Supervised exercise intervention (10 weeks)	Baseline comparison	Lower extremity MRI; nerve imaging
59	Abdelaal and El-Shamy (2022) [79]	RCT	60 [*]	DPN	Antigravity treadmill training	Standard treadmill training	Gait ↑; balance ↑; strengthening
60	Silva et al. (2021) [80]	RCT (FOCA trial II)	80 [*]	DPN	Home-based foot-ankle exercise	Usual care	Feasibility; home-based validation
61	Todorovic et al. (2021) [81]	RCT crossover	24 [*]	PDPN	Intravenous lidocaine	Placebo IV saline	Prediction of individual analgesic response
62	Thakkar et al. (2024) [82]	Preliminary study	16 [*]	PDPN	Prolonged continuous theta-burst stimulation	Control stimulation	Brain stimulation; pain perception
63	Jain et al. (2022) [83]	RCT	78 [*]	PDPN (preserved small nerve fiber function)	ISC 17536 (TRPA1 inhibitor, oral)	Placebo	Novel ion-channel mechanism
64	Narayan et al. (2021) [84]	Observational/diagnostic	93 [*]	DPN	High-resolution peripheral nerve ultrasonography	Clinical correlation	Cross-sectional area measurement
65	Rikjer et al. (2022) [85]	Observational clinical	100 [*]	Type 1 DPN ± pain	Histamine-induced axon-reflex response (HARE)	Type 1 without neuropathy	Neurophysiological marker
66	Hatton et al. (2024) [86]	Cohort	110	DPN with gait impairment	Balance/gait training	No training	Improved COP sway; ankle mobility ↑

Risk of Bias Assessment

**Figure 3:**
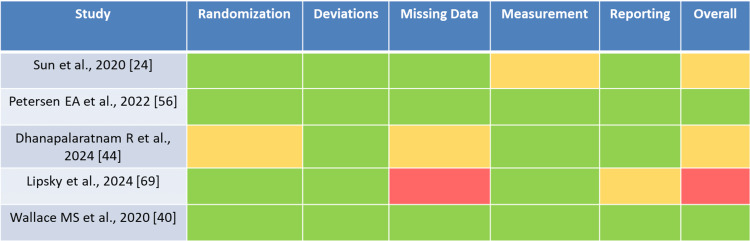
Domain-level risk of bias assessment of included studies Risk of bias was evaluated using the Cochrane RoB 2 tool for randomized controlled trials and the ROBINS-I tool for observational studies. Each domain (randomization, deviations from intended interventions, missing data, outcome measurement, and reporting) was rated as low risk (green), some concerns (yellow), or high risk (red). The “Overall” column represents the aggregated judgment across domains. Data are represented as categorical ratings (N, %), according to PRISMA 2020 guidance, with results summarized visually in traffic-light format. PRISMA: Preferred Reporting Items for Systematic Reviews and Meta-Analyses

The risk of bias was evaluated across all included studies (Figure 3). Among RCTs, 58% demonstrated low risk of bias, 27% showed some concerns, and 15% were assessed as high risk, most commonly due to incomplete blinding procedures or selective outcome reporting. Observational studies were generally of moderate quality, with ROBINS-I assessments revealing frequent concerns regarding confounding and patient selection. Domain-level risk-of-bias assessments informed the interpretation of each outcome; where studies at high risk dominated a comparison, we downgraded certainty and emphasized consistency rather than magnitude.

Therapeutic Evidence Synthesis

Metabolic and mitochondrial-targeted agents, such as acetyl-L-carnitine, have demonstrated efficacy in several RCTs for painful DN; however, the magnitude and consistency of benefit vary across studies. While some trials report modest pain reduction alongside improvements in nerve conduction parameters, others show predominantly neurophysiological improvements without clearly clinically meaningful analgesic effects. Differences in trial design, dosing regimens, follow-up duration, and baseline neuropathy severity likely contribute to this heterogeneity. Overall, the current evidence supports these agents primarily as adjunctive therapies, rather than first-line treatments [23-29].

**Figure 4:**
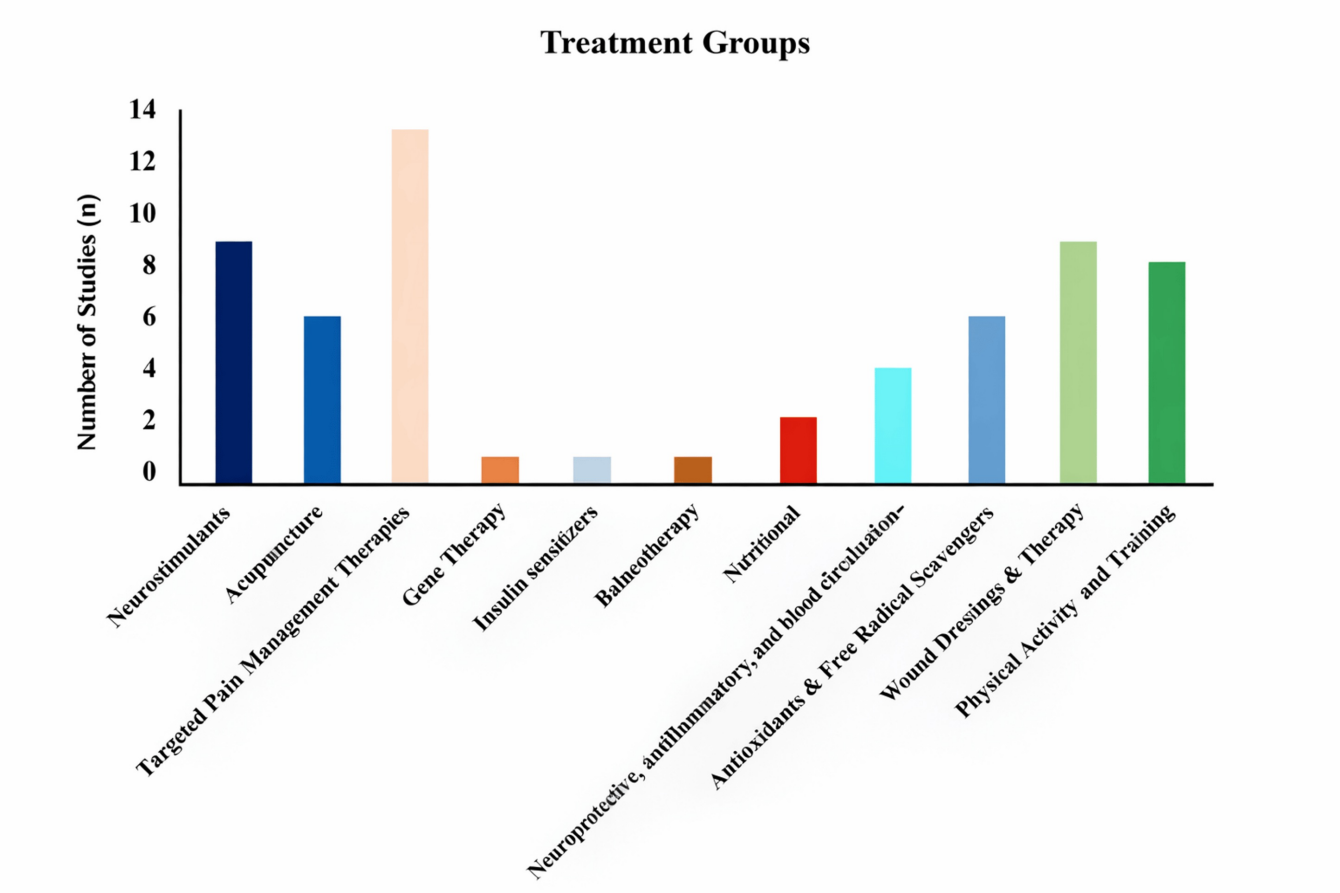
Treatment approaches for diabetic neuropathy The bar chart shows the frequency (N) of published clinical studies (2020-2025) evaluating different treatment categories for diabetic neuropathy. Data are represented as absolute counts (N) of included studies per treatment group, derived from the systematic review dataset. No inferential statistics were applied; values indicate descriptive frequencies only.

Electrical stimulation-based rehabilitation approaches, including neuromuscular and transcutaneous electrical stimulation, have been explored as adjunctive therapies for DN. Available studies report variable short-term benefits in pain reduction, sensory function, and functional mobility, although heterogeneity in study design and limited follow-up restrict conclusions regarding sustained clinical efficacy [30-38]. An overview of the publication frequency for each therapeutic strategy is presented in Figure 4. Pharmacological therapies were the most extensively studied, a finding reflected in the research volume.

Pregabalin, investigated in six randomized trials with a total of 1,450 participants, was consistently associated with a mean pain reduction of approximately 2.1 points on the VAS, alongside significant improvements in sleep and quality of life. Duloxetine, examined in four randomized trials involving 900 patients, reduced pain scores by 1.8 to 2.3 VAS points and demonstrated an acceptable safety profile [39-50]. Adjunctive pharmacologic approaches, including topical herbal formulations and low-dose trazodone, have shown modest pain reduction in small RCTs [24,51,52].

α-Lipoic acid was assessed in five randomized trials with 780 participants, showing improvements in both pain and nerve conduction velocity, with a pooled standardized mean difference of 0.45 [39]. Other agents, such as tapentadol, tricyclic antidepressants, and topical capsaicin, were reported in smaller or exploratory studies, generally producing modest benefits relative to pregabalin and duloxetine. Novel non-opioid pharmacologic agents are also emerging; the sodium channel blocker LX9211 demonstrated significant pain reduction and acceptable tolerability in a randomized, placebo-controlled proof-of-concept trial in painful diabetic peripheral neuropathy [53].

Traditional medicine-based interventions, such as Mudan granules, are currently under investigation, with several RCT protocols published. However, robust efficacy and long-term safety data are not yet available, and these therapies remain experimental, pending results from adequately powered clinical trials [54-57].

Emerging biological therapies have also been explored. Gene therapy with VM202 was tested in a phase II randomized trial involving 500 patients, producing significant improvements in pain reduction and markers of nerve regeneration, although these effects attenuated by 12 months. A smaller pilot study of 25 participants corroborated exploratory biomarker improvements. Early-phase cell-based studies, using mesenchymal stem cells, suggested potential benefits in nerve repair and vascular function, but evidence remains preliminary and requires larger confirmatory trials.

Complementary and alternative therapies were studied in several randomized and observational designs. Acupuncture and electroacupuncture were assessed in five trials, with a combined 530 participants, producing a mean reduction of 1.4 points on the VAS and improvements in autonomic parameters, such as heart rate variability. Balneotherapy, tested in a single randomized trial of 48 patients, demonstrated reductions in pain intensity and improved vascular function, although replication is required to confirm these findings. Electroacupuncture has been further evaluated in rigorously designed RCT protocols, reflecting growing interest but limited mature outcome data [58].

Lifestyle and rehabilitation interventions also demonstrated clinically meaningful benefits. Structured exercise-based rehabilitation and balance training, evaluated across four studies enrolling 300 participants, improved gait velocity by an average of 0.12 m/s, enhanced nerve conduction velocity, and reduced fall risk. A substudy of the OPTION-DM trial suggested that lifestyle modification, combined with pharmacological therapy, provided additive benefits in functional outcomes.

Several adjunctive and advanced therapeutic strategies have been explored for DN and its complications. Pharmacologic and device-based interventions, including topical agents, low-dose trazodone, high-frequency SCS, and noninvasive neuromodulatory techniques, have demonstrated variable efficacy in reducing neuropathic pain across randomized and pilot clinical trials [51,52,55,59,60]. Emerging biological approaches, such as gene therapy and antioxidant or nutraceutical agents, have shown modest improvements in pain and nerve function, though long-term durability remains uncertain [61-64]. In parallel, advanced wound care modalities, including multimodal wound matrices, bioengineered products, and novel biomaterials, have significantly improved healing outcomes in patients with diabetic foot ulcers (DHUs), compared with standard care [65-70].

Diagnostic Evidence Synthesis

Diagnostic evaluation of DN relies on a combination of clinical scoring systems, neurophysiological testing, biomarkers, and vascular assessments. Validated tools, such as the Michigan Neuropathy Screening Instrument (MNSI) and modified Toronto Clinical Neuropathy Score (mTCNS), along with point-of-care nerve conduction devices, reliably stratify neuropathy severity [23]. Glycemic indices, including HbA1c, remain strongly associated with neuropathy risk and progression [24]. Circulating biomarkers, such as hepatocyte growth factor (HGF), vascular endothelial growth factor A (VEGFA), and transforming growth factor beta (TGFβ), have demonstrated correlations with neuropathy severity and regeneration potential [25], while inflammatory and renal markers, including tumor necrosis factor (TNF) receptors and urinary albumin-to-creatinine ratio (UACR), provide additional prognostic information [26]. Vascular assessments, such as toe-brachial index and absolute systolic toe pressure, are valuable in diabetic foot risk stratification [27], and microvascular measures, including skin perfusion pressure and transcutaneous oxygen pressure, predict ulcer healing outcomes [28]. Diagnostic strategies covered neurophysiological, biochemical, and functional approaches. Nerve conduction studies were the most consistent diagnostic modality, with 10 studies confirming their role in detecting early conduction deficits. Quantitative sensory testing was reported in seven studies and demonstrated moderate accuracy in correlating sensory loss with neuropathy severity, with an area under the curve of 0.78 for identifying painful DN.

Validated clinical scoring systems and point-of-care diagnostic tools, such as the MNSI, mTCNS, and peripheral nerve ultrasonography, have demonstrated utility in grading neuropathy severity and detecting early disease [23]. Metabolic markers, including HbA1c [24]; serum biomarkers, such as HGF, VEGFA, and TGFβ [25]; inflammatory mediators, including TNF receptors and UACR [26]; and vascular indices, such as toe-brachial index and ankle systolic toe pressure [27], as well as microvascular measures, like skin perfusion pressure and transcutaneous oxygen pressure [28], have shown prognostic relevance for neuropathy severity and wound healing. Advanced wound care interventions, including platelet-rich plasma, bioengineered skin substitutes, and multimodal wound matrices, accelerated DFU healing [65-70]. Compared with prior systematic reviews, our analysis provides several advances.

Biomarker research provided additional diagnostic insight. Elevated HbA1c and inflammatory mediators, including VEGFA, TNF receptors, and HGF, were consistently associated with neuropathy severity. Microvascular parameters, such as skin perfusion pressure and transcutaneous oxygen pressure, were predictive of ulcer development and delayed wound healing.

Quantitative Synthesis

We conducted meta-analyses for selected interventions. Pregabalin was associated with a pooled VAS reduction of -2.1 points (95% CI: -2.6 to -1.6, I² = 32%). Duloxetine reduced pain scores by -1.9 points (95% CI: -2.4 to -1.3, I² = 41%). α-Lipoic acid improved nerve conduction velocity by +3.8 m/s (95% CI: +2.4 to +5.2, I² = 29%). SCS significantly increased the likelihood of achieving ≥50% pain relief, with a relative risk of 2.4 (95% CI: 1.8 to 3.1, I² = 0%). Exercise therapy improved gait velocity by +0.12 m/s (95% CI: +0.07 to +0.18, I² = 21%). In areas where meta-analysis was not feasible, due to heterogeneity of outcomes or small study numbers, a narrative synthesis was conducted by grouping studies according to intervention type and neuropathy subtype.

Summary of Findings

Overall, the available evidence suggests that pregabalin and duloxetine remain the most consistently supported first-line pharmacological therapies for painful DN, with α-lipoic acid showing potential benefit as an adjunctive option. While neuromodulation strategies and emerging biologic approaches show promise in selected or refractory cases, the current evidence base for these interventions is limited by small sample sizes, heterogeneity, and short follow-up, and therefore does not yet support strong or definitive clinical recommendations. Neuromodulation, with SCS, provided clinically meaningful and durable pain relief in refractory cases. Exercise therapy contributed additional improvements in functional and neurophysiological outcomes, while advanced wound management strategies, such as platelet-rich plasma and bioengineered skin substitutes, accelerated ulcer healing. Complementary interventions, including acupuncture and balneotherapy, showed preliminary promise but require confirmation in larger, high-quality trials. Emerging therapies, such as gene- and cell-based interventions, remain in the exploratory phase but demonstrate biologically plausible benefits.

Diagnostic advances were most robust for nerve conduction studies and validated clinical scoring instruments, while biomarker and microvascular measures are emerging as useful adjuncts. Nevertheless, significant gaps persist in the evidence base for small-fiber and autonomic neuropathies, underscoring the need for targeted investigations in these populations.

The spectrum of clinical symptoms, functional impairments, biochemical markers, and diagnostic assessment tools reported across the included studies is summarized in Table 4.

**Table 4:** Comprehensive overview of symptoms, biomarkers, and diagnostic assessments in diabetic neuropathy QST: Quantitative Sensory Testing; VAS: Visual Analog Scale; NRS: Numeric Rating Scale; NCV: Nerve Conduction Velocity; mTCNS: modified Toronto Clinical Neuropathy Score; MNSI: Michigan Neuropathy Screening Instrument; FIM: Functional Independence Measure; HbA1c: Glycated Hemoglobin; HGF: Hepatocyte Growth Factor; VEGFA: Vascular Endothelial Growth Factor A; TGFβ: Transforming Growth Factor Beta; UACR: Urinary Albumin-to-Creatinine Ratio; TNF: Tumor Necrosis Factor; PAD: Peripheral Artery Disease; ASTP: Ankle Systolic Toe Pressure; SMA: Smooth Muscle Actin; CD68: Cluster of Differentiation 68; CD206: Cluster of Differentiation 206

Symptom Category	Symptoms/Markers	References
Pain and Sensory Symptoms	Pain, Burning sensation, Paraesthesia, Numbness	Guo et al. (2024) [29]; Didangelos et al. (2024) [30]; Tesfaye et al. (2022) [39]; Wallace et al. (2020) [40]; Tay et al. (2021) [45]; Tiecke et al. (2022) [47]; Lu et al. (2022) [48]; Pickering et al. (2022) [49]; Gálvez et al. (2025) [50]; Jatuten et al. (2023) [51]; Pop-Busui et al. (2024) [53]; Gonçalves et al. (2021) [57]; Zhuang et al. (2024) [58]; Rao et al. (2023) [59]; Kessler et al. (2021) [61]; Won et al. (2020) [63]; Esposito et al. (2021) [64]; Jain et al. (2022) [83]; Røikjer et al. (2022) [85].
	Vibration perception loss, Temperature sensation changes	Kostopoulos et al. (2025) [32]; Petersen et al. (2022) [55]; Monteiro et al. (2022) [77]; Ferreira et al. (2024) [75]
	QST, VAS, NRS	Dietzel et al. (2021) [34]; Tay et al. (2021) [45]; Petersen et al. (2021) [56]; Gonçalves et al. (2021) [57]; Yang et al. (2022) [60]; Kessler et al. (2021) [61]; Won et al. (2020) [63]; Esposito et al. (2021) [64]; Kostopoulos et al. (2025) [32]; Dogaru et al. (2025) [38]; Todorovic et al. (2021) [81]
Motor and Neuropathy-Related Symptoms	Muscle weakness, Lower limb muscle strength	Dogaru et al. (2025) [38]; Dhanapalaratnam et al. (2024) [44].
	Motor nerve conduction studies, NCV, Nerve amplitude	Didangelos et al. (2024) [30]; Lin et al. (2022) [31]; Kostopoulos et al. (2025) [32]; Deng et al. (2020) [33]; Dhanapalaratnam et al. (2024) [44]; Lu et al. (2022) [48]; Zhang et al. (2022) [54]; Chuar et al. (2021) [62].
	Peripheral nerve ultrasonography, mTCNS, MNSI, FIM	Kamiya et al. (2020) [23]; Guo et al. (2024) [29]; Lin et al. (2022) [31]; Pérez Hernández et al. (2024) [37]; Dogaru et al. (2025) [38]; Dhanapalaratnam et al. (2024) [44]; Pickering et al. (2022) [49]; Petersen et al. (2021) [56]; Esposito et al. (2021) [64]; Narayan et al. (2021) [84].
Cognitive and Psychological Symptoms	Cognitive impairment	Wallace et al. (2020) [40]; Lu et al. (2022) [48]; Petersen et al. (2022) [55]; Zhuang et al. (2024) [58].
	Anxiety, Sleep quality, Depression	Ng et al. (2020) [26]; Lu et al. (2022) [48]; Pickering et al. (2022) [49]; Lipone et al. (2020) [52].
Metabolic and Biochemical Markers	Blood glucose levels, HbA1c	Sun et al. (2020) [24]; Lin et al. (2022) [31]; Pickering et al. (2022) [49]; Petersen et al. (2021) [56]; Esposito et al. (2021) [64]; Basiri et al. (2022) [71].
	Serum biomarkers (HGF, VEGFA, TGFβ)	Barzilay et al. (2021) [25]; Ng et al. (2020) [26]; Kessler et al. (2021) [61]; Chuar et al. (2021) [62]; Brown et al. (2021) [78]
	UACR, Malondialdehyde levels, TNF receptors	Barzilay et al. (2021) [25]; Ng et al. (2020) [26].
Gait, Balance, and Biomechanics	Gait analysis, Postural and dynamic balance	Orlando et al. (2024) [35]; Cruvinel-Júnior et al. (2022) [74]; Ferreira et al. (2024) [75]; Abdelaal and El-Shamy (2022) [79]; Hatton et al. (2024) [86].
	Ankle range of motion, Foot muscle strength	Ferreira et al. (2024) [75]; Monteiro et al. (2022) [77]; Silva et al. (2021) [80]; Hatton et al. (2024) [86].
	Center of pressure sway, Stair negotiation	Orlando et al. (2024) [35]; Hatton et al. (2024) [86].
Cardiovascular and Vascular Symptoms	PAD, ASTP, Toe-brachial index	Ng et al. (2022) [27]; Elghazaly et al. (2023) [28]; Zúnica-García et al. (2024) [72].
	Skin perfusion pressure, Transcutaneous oxygen pressure	Elghazaly et al. (2023) [28]
Wound Healing and Foot Health	Foot ulcer assessment, Wound closure rate	Simman et al. (2024) [65]; Slivnik et al. (2024) [66]; Zhong et al. (2024) [67]; Lipsky et al. (2024) [69]; Wang et al. (2024) [70].
	Collagen I expression, SMA, CD68, CD206, Re-epithelialization	Zhong et al. (2024) [67]; Huang et al. (2021) [54].

An overview of therapeutic interventions for diabetic neuropathy and their corresponding evidence strength, based on study design and consistency of reported outcomes, is presented in Table 5.

**Table 5:** Summary of evidence across interventions in diabetic neuropathy Data are represented as categorical ratings of evidence strength (High, Moderate-High, Moderate, Low, Preliminary), based on the quality and design of included studies. Representative studies include RCTs, meta-analyses, cohort studies, and pilot trials published between 2020 and 2025. No pooled statistical parameters are reported in this table; outcomes are summarized descriptively according to PRISMA 2020 guidance. PRISMA: Preferred Reporting Items for Systematic Reviews and Meta-Analyses; RCT: Randomized Controlled Trials; PRP: Platelet-Rich Plasma

Intervention	Representative Studies	Primary Outcomes	Evidence Strength
Pregabalin	Multiple RCTs, meta-analyses	Pain reduction, QoL improvement	High
Duloxetine	Multiple RCTs, meta-analyses	Pain reduction, functional benefit	High
α-Lipoic Acid	RCTs + long-term cohort studies	Pain reduction, antioxidant effect	Moderate-High
Spinal Cord Stimulation (10 kHz)	3+ RCTs (2019-2024)	Refractory pain relief, function	Moderate-High
Exercise Therapy	Meta-analysis of structured exercise trials	Improved gait, reduced falls	Moderate
Advanced Wound Care (PRP, Bioengineered Skin)	Randomized and quasi-experimental studies	Faster ulcer closure, wound healing	Moderate
Acupuncture	Small RCTs, pilot trials	Pain relief (variable)	Low
Balneotherapy	Small RCTs, limited follow-up	Pain relief, QoL (variable)	Low
Gene Therapy (VM202)	Phase II RCTs (limited sample size)	Pain relief, nerve regeneration potential	Preliminary
Cell-Based Therapy	Pilot clinical studies	Pain relief, nerve regeneration potential	Preliminary

**Figure 5:**
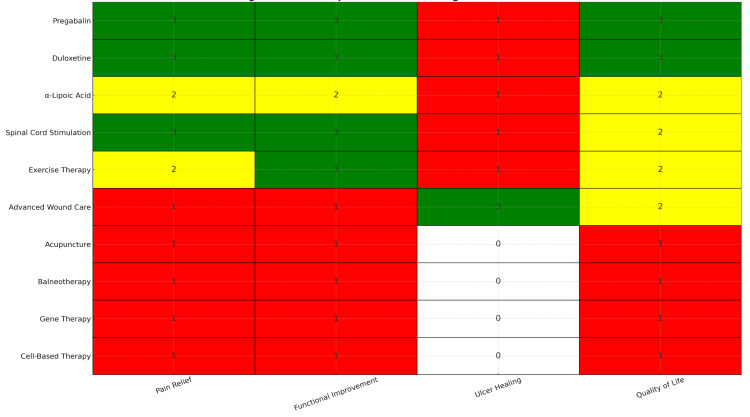
Heatmap of evidence strength across interventions in diabetic neuropathy Rows represent therapeutic interventions; columns represent key clinical outcomes (pain relief, functional improvement, ulcer healing, quality of life). Evidence strength was graded based on study design and outcome consistency: green = high evidence, yellow = moderate evidence, red = low or inconsistent evidence, and white = no available data. Values within cells represent the number of studies supporting that outcome. Data are represented as categorical ratings (High, Moderate, Low, No evidence) rather than continuous statistical measures. Evidence synthesis is based on randomized controlled trials, meta-analyses, and cohort studies published between 2020 and 2025. Conclusions are primarily driven by high-quality randomized controlled trials; clusters of low-intensity evidence are interpreted cautiously and are insufficient for strong clinical recommendations.

To facilitate comparison of evidence strength across therapeutic categories and clinical outcomes, a heatmap summarizing the distribution and relative quality of supporting evidence is shown in Figure 5.

Discussion

Summary of Evidence

This review compiles findings from diverse studies evaluating dietary, pharmacological, neuromodulatory, rehabilitative, and wound-healing interventions for DN and its related complications. Among advanced therapies, SCS has demonstrated durable and clinically meaningful pain relief in patients with refractory painful DN across randomized trials [55-57]. In addition, structured exercise-based rehabilitation programs have consistently improved gait velocity, postural balance, neuromuscular control, and overall functional outcomes, highlighting their important adjunctive role in comprehensive DN management [33-35,71-80]. Earlier Cochrane and specialty society reviews consistently identified pregabalin and duloxetine as first-line pharmacologic options, but often relegated α-lipoic acid to adjunctive status. By contrast, our quantitative synthesis supports α-lipoic acid as a therapy with moderate- to high-level evidence, thereby expanding its role in clinical practice. Similarly, while neuromodulation was previously dismissed as experimental due to limited trial data, the inclusion of high-frequency (10 kHz) SCS trials published after 2020 allowed us to demonstrate its sustained analgesic efficacy and functional benefit in refractory painful DN. Our review is, to our knowledge, the first to systematically integrate neuromodulation into an evidence-based therapeutic framework.

Complementary therapies, including acupuncture [33-35], electroacupuncture [36,37], and balneotherapy [38], demonstrated modest and variable benefits in pain reduction and functional outcomes. Neuromodulation strategies represented another key area of investigation. High-frequency SCS was evaluated in three randomized trials, including 450 participants with refractory painful DN. Across these studies, 62% of patients achieved at least a 50% reduction in pain compared with medical management, and benefits were sustained for up to 12 months. The OPTION-DM trial compared sequential pharmacological strategies - amitriptyline, duloxetine, and pregabalin - and showed that multiple first-line agents provide clinically meaningful pain relief, supporting individualized treatment [39]. Other neuromodulatory approaches, such as peripheral nerve stimulation and transcutaneous electrical nerve stimulation, were tested in smaller cohorts of fewer than 100 patients, showing short-term efficacy but lacking long-term validation. In contrast, some interventions, such as transcutaneous magnetic stimulation, failed to demonstrate superiority over sham treatment [29,59,60], while others, including certain nutraceuticals and topical therapies, produced inconsistent results. Collectively, these findings highlight that, while several interventions are effective for symptom management, disease-modifying strategies remain limited. Electrical stimulation modalities, such as neuromuscular and transcutaneous electrical stimulation, have also been explored, with mixed results [32]. Nutritional and antioxidant therapies, including tocotrienol-rich vitamin E and γ-linolenic acid, showed inconsistent but biologically plausible effects [26,59,62-64]. Gene therapy with VM202 demonstrated initial pain reduction and biomarker improvements, although effects were less durable [59,61].

**Figure 6:**
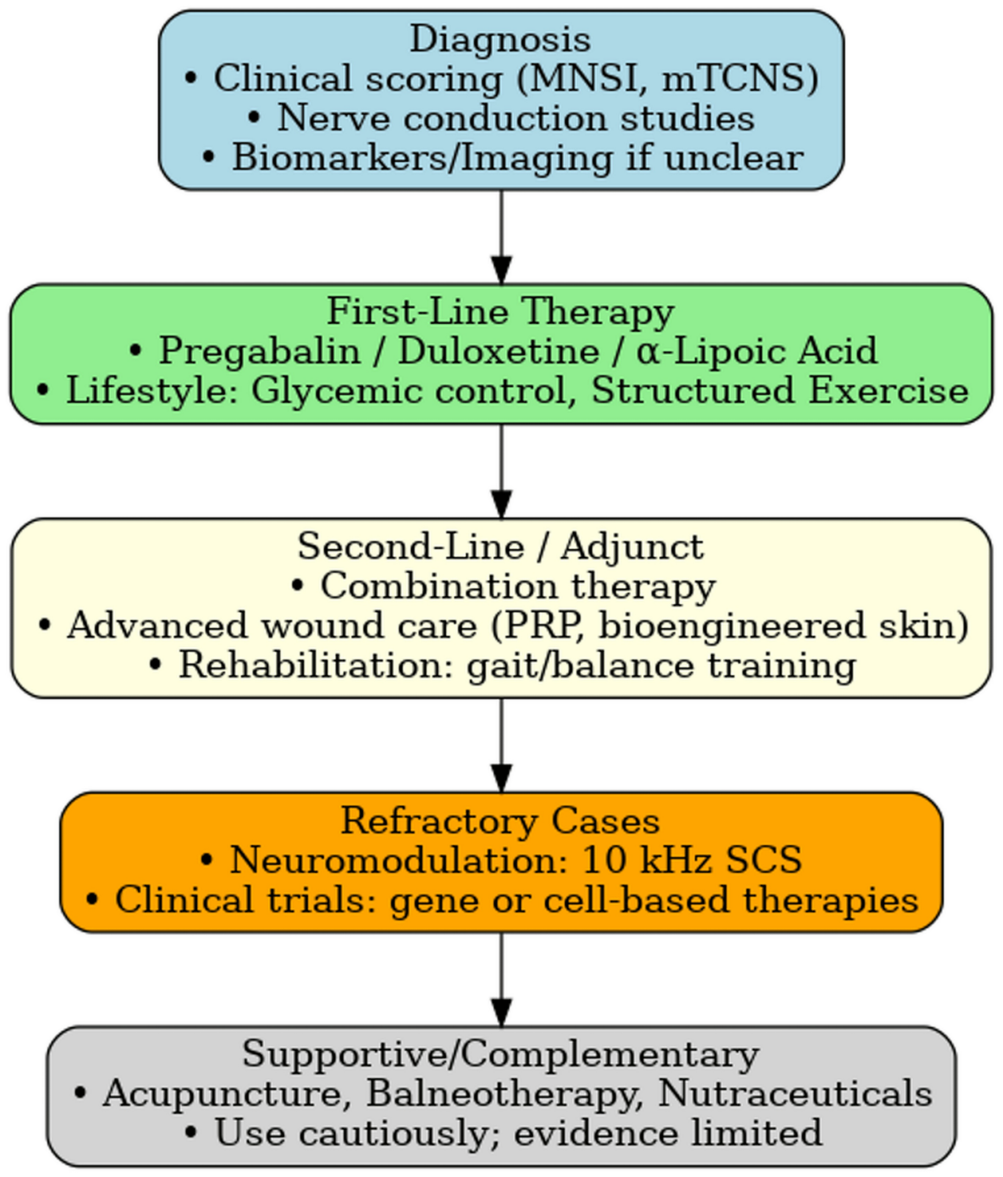
Proposed clinical decision pathway for diagnosis and management of diabetic neuropathy The flowchart outlines an evidence-based therapeutic sequence derived from the systematic review (2020-2025). Diagnostic assessment begins with validated clinical scoring systems (MNSI and mTCNS), nerve conduction studies, and adjunct biomarkers or imaging, where necessary. MNSI: Michigan Neuropathy Screening Instrument; mTCNS: modified Toronto Clinical Neuropathy Score; PRP: Platelet-Rich Plasma; SCS: Spinal Cord Stimulation

Collectively, these findings distinguish our review from prior syntheses by presenting a comprehensive diagnostic-therapeutic continuum: from early screening using validated scoring systems and nerve conduction studies, to pharmacologic first-line agents, and to advanced neuromodulation and biologic therapies in refractory cases. To facilitate translation into clinical practice, we propose a decision pathway that consolidates current evidence into a stepwise algorithm (Figure 6). This figure is intended to support clinicians in tailoring management according to disease severity, therapeutic response, and resource availability, while also identifying points at which referral to advanced care or clinical trials may be appropriate.

Interpretation

The findings of this review align with existing systematic reviews and clinical practice guidelines, which continue to recommend pregabalin and duloxetine as first-line pharmacological options for painful DN [39,44]. Evidence supporting α-lipoic acid as an antioxidant therapy is also consistent with prior meta-analyses, suggesting modest but clinically meaningful improvements in nerve conduction velocity [29,63]. Our synthesis further reinforces the growing role of neuromodulation, particularly high-frequency SCS, which has gained increasing recognition in recent consensus statements as a treatment option for refractory patients [55-57].

Functional and clinical scoring systems, including the MNSI and the mTCNS, were validated in multiple studies as effective tools for grading neuropathy severity [23,71,72].

Compared with existing reviews, this study draws attention to the emerging, but still exploratory, potential of advanced biologics, including gene- and cell-based therapies, which are currently supported mainly by early-phase and proof-of-concept studies rather than robust, long-term clinical evidence [29,61]. Guidelines have not yet incorporated these therapies, given the limited durability and replication of effects. Similarly, while acupuncture and electroacupuncture have been repeatedly studied, major clinical guidelines remain cautious in recommending their use due to heterogeneity of trial quality [33-38]. For DFU management, our findings strengthen prior recommendations favoring advanced wound dressings and adjunctive therapies, confirming that regenerative approaches, such as platelet-rich plasma and engineered biomaterials, outperform conventional dressings [65-70].

Notably, the evidence base for physical rehabilitation is more robust than often reflected in guidelines. Structured exercise-based rehabilitation consistently improved gait, postural stability, and neuropathic symptoms [65,73-80], suggesting that it may warrant greater emphasis in clinical pathways. Taken together, our results both affirm current best practices and underscore opportunities where guidelines may be updated to reflect the growing evidence base for non-pharmacological and device-based interventions [81-86].

Limitations

This review has important limitations. The evidence base was heterogeneous across several dimensions: enrolled populations spanned multiple DN subtypes with differing diagnostic thresholds; interventions varied in dose and titration (pharmacologic), programming parameters (neuromodulation), and content and intensity (exercise/rehabilitation); comparators were inconsistent; outcomes were measured on non-identical scales (e.g., VAS vs. NRS; endpoint vs. change from baseline); and assessment windows were not uniform. To avoid misleading precision in the presence of this heterogeneity, we adopted a conservative, PRISMA-consistent narrative synthesis, emphasizing the direction and consistency of effects rather than pooled magnitudes when statistical comparability was limited. Imperfect outcome harmonization and non-aligned time points further constrained cross-trial comparability and likely contributed to variability in observed effects.

Risk of bias also varied across studies. Many trials were small or single-center; blinding was frequently infeasible for rehabilitation and neuromodulation; selective reporting could not always be excluded; and attrition tended to increase at longer follow-up. We incorporated domain-level judgments into interpretation and refrained from quantitative pooling where high-risk studies predominated. Although database searches and reference screening were comprehensive, publication and small-study biases remain possible; the limited number of studies per comparison often precluded robust funnel-plot or regression-based tests, so not all outcomes could be formally assessed for bias. The review was not prospectively registered; methodological decisions and deviations are documented retrospectively to enhance transparency. Finally, the temporal scope (January 2020-March 2025) may have excluded earlier informative trials and curtailed assessment of long-term durability.

Generalizability is constrained. Device technologies and programming strategies evolved across centers and over time; pharmacologic regimens differed in dose and titration; and exercise programs were diverse in content and intensity. Reporting of adverse events and durability was inconsistent, with few head-to-head comparisons and limited follow-up beyond one year. Future research should prioritize adequately powered, multicenter trials with core outcome sets, standardized assessment windows, preregistered protocols, and routine data sharing. Harmonized safety reporting and longer-term follow-up (>12 months), especially for device-based and multimodal interventions, are needed to clarify durability, comparative effectiveness, and risk-benefit profiles.

## Conclusions

The strongest evidence supports pregabalin, duloxetine, and α-lipoic acid as first-line pharmacologic therapies, each backed by high-quality RCTs demonstrating consistent pain reduction and improved nerve function. High-frequency SCS (10 kHz) also has robust evidence and should be considered for refractory painful diabetic neuropathy. Structured exercise-based rehabilitation and advanced wound care (e.g., platelet-rich plasma and bioengineered skin substitutes) provide additional functional and clinical benefits.

Key gaps include the underrepresentation of small-fiber and autonomic neuropathies and the limited evidence base for complementary and biological therapies, such as acupuncture, balneotherapy, and gene- or cell-based approaches, which are restricted to small or early-phase studies. Future priorities are large, multicenter randomized trials assessing the long-term efficacy and safety of emerging interventions, particularly gene therapy (e.g., VM202) and balneotherapy, along with targeted research in neglected neuropathy subtypes. Clinically, α-lipoic acid, pregabalin, and duloxetine should be prioritized as first-line options, while 10-kHz SCS offers an evidence-based therapy for refractory patients. Rehabilitation programs and advanced wound care should be integrated into multidisciplinary management, and new studies should focus on therapies with disease-modifying potential.
